# Perspective of material evolution Induced by sinusoidal reflex charging in lithium-ion batteries

**DOI:** 10.1016/j.heliyon.2024.e30471

**Published:** 2024-05-04

**Authors:** Huang K. David, Po-Tuan Chen, Wei-Mon Yan, Thangavel Sangeetha, Cheng-Jung Yang

**Affiliations:** a Department of Vehicle Engineering, National Taipei University of Technology, Taipei 10608, Taiwan; b Department of Energy and Refrigerating Air-Conditioning Engineering, National Taipei University of Technology, Taipei, 10608, Taiwan; c Research Center of Energy Conservation for New Generation of Residential, Commercial, and Industrial Sectors, National Taipei University of Technology, Taipei, 10608, Taiwan; d Program in Interdisciplinary Studies, National Sun Yat-sen University, Kaohsiung, Taiwan

**Keywords:** Lithium-ion batteries, Constant current-constant voltage charging, Sinusoidal reflex charging, Impedance, Solid electrolyte interface, Secondary batteries

## Abstract

**Background:**

Lithium-ion batteries are globally prominent and extensively employed alternative energy sources with decisive applications. In depth understanding of influences of various charging and discharging cycles on electrode materials and life span of these batteries is critical as cycle-life and safety of lithium-ion batteries are closely related crystallinity of electrode materials. This study is a detailed investigation endeavor in observing the degree of damage to electrode materials under multiple charging and discharging cycles.

**Method:**

ology: A constant current-sinusoidal reflex charging method (CC-Sinusoidal) was implemented to charge commercial cathode Lithium cobalt oxide (LiCoO_2_) electrodes and anode graphite electrodes in comparison to the conventional charging method of constant current-constant voltage (CC-CV). After 100, 300, and 500 cycles of charging and discharging, EIS, SEM, XRD, and Raman spectroscopies were used to compare the degree of electrode damage caused by different charging methods.

**Significant outcomes:**

The structure of positive LiCoO_2_ electrode of the battery was observed to be stable, with no significant change in both the charging methods after 500 cycles. The use of CC-CV charging method had caused severe damages to graphite electrode with generation of solid electrolyte interface (SEI) films. The CC-Sinusoidal charging method had maintained the electrode material in a relatively ideal state.

## Highlights

• Multiple charging and discharging cycles of Lithium ion batteries were conducted.
• Constant current-sinusoidal and current-constant voltage methods were compared.
• Impacts on graphite anode and LiCoO_2_ cathode electrodes was investigated.
• The investigation was done for 100, 300, and 500 cycles.
• EIS, SEM, XRD, and Raman spectroscopy techniques evaluated the electrode damages.

## 1 Introduction

With rapid depletion of global energy resources and environmental deterioration, discovery and development of innovative energy storage devices is being considered highly significant. Lithium-ion battery (LiBs) is one such state-of-the-art technology which has received immense attention and research development in the last three decades. Their enormous progress as secondary batteries have effectively solved energy storage applications, making them inevitable in smart grids, microgrids, intelligent electronic devices, and electric vehicles and thereby contributing to the development in renewable energy production, carbon emissions reduction, energy storage, low self-discharge rate, high energy density, safe performance, economic benefits and numerous other advantages. These batteries require repeated charge and discharge cycles, that reduce their operational life, and significant modifications in the charging techniques and charging management systems of these batteries can greatly aid in reducing the shortcomings and improve their state of health (SOH) [1]**.** Immense research emphasis has been placed on the scaling up of these exciting devices, as they have certain bottlenecks like low power density, poor electrochemical performance, limited recycling, cycle-life aging caused due to charging as they limit their further applications. Many charging methods like Constant Current (CC), Constant Voltage (CV), Pulse Charging (PC), Negative Pulse Charging (NPC), Open and closed loop charging have been proposed for these batteries [2].

At present, the regularly used and widely considered charging stratagem for lithium-ion batteries in the market is the constant current-constant voltage (CC-CV) charging method. This can be simply explained as follows: When a LiB is connected to the charger, the CC mode is commenced to maintain electric current flow at a constant level, whereas the voltage within the battery keeps escalating and reaches a threshold limit with time. Later, the charging is transformed into the CV mode to avoid overvoltage and safety management. The voltage then gets sustained at a steady level and the amount of the current gets reduced. Finally, when the current attains the cut-off level, charging is determined as complete [3]. The advantages of CC-CV charging method are current size control, fulfilment of customer requirements and most importantly fast charging (with a large number of C-rates), thus shortening a large amount of charging time to compensate for the CV, which requires a long time for fully charging the battery [4,5]. But it also has certain drawbacks like higher and quicker degradation with reduced charging time. In order to overcome these issues and improve the lifespan of batteries due to the problem of battery aging caused by charging, an innovative sinusoidal wave charging strategy was successfully proposed [6,7]. This method had a uniform and fast battery charging ability [8], which also greatly reduced the lithium precipitation and extended the battery life [9].

Electrodes are significant components in LiBs, and their advantageous characteristics will regulate trouble free performance of the batteries. Electrodes with great mechanical strength, extra ordinary corrosion resistance, large surface to dimension ratios and fully exposed active surfaces are generally preferred. In order to pursue fast charging and use a larger C-rate for charging, lithium ions quickly embed and insert between the two electrodes, causing the interface of the electrolyte and electrode to be unstable. Due to the decomposition of the lithium-electrolyte complex during charging, the passivation film of the solid electrolyte interface (SEI) on the electrode thickens, accelerating battery aging, increasing impedance, and reducing battery capacitance, and even the rapid insertion of lithium ions into the negative electrode, resulting in the detachment of the electrode structure. Lithium-ion batteries may experience a decrease in capacitance, especially irreversible capacitance loss. Reduction of recyclable lithium ions in batteries, damage to electrode materials, and increase in battery impedance [[10], [11], [12], [13]]. Various electrode materials have been employed in LiBs as anode and cathode electrodes. Li metal [14]; silicon based anodes [15]; polymeric materials and tin oxide [16] etc. Nevertheless, carbon-based graphite has been highly commercialized as anode electrode owing to its good reversibility, high conductivity and affordable cost [17]. It is still the most common anode material used in LiBs (>95 %) and dominates the market due to its comparatively greater energy density combined with low cost and availability [18]. Likewise, diverse materials have been utilized as cathode electrodes in LiBs like crystalline state oxide or phosphate-based materials as they guarantee enhanced Li-ion mobility. Potassium and sulfur doped with Li–Mg spinals had heightened ion agility [19]. The grain boundaries in oxide cathodes can reduce the ion-conductivity, so oxide glass materials were used as LiB cathodes. Doping of these glass materials with metal oxides will improve the electrochemical performance [20]. High coulombic efficiencies can be acquired by iron-based cathode electrode materials [21]. LiCoO_2_ is considered beneficial than other cathode materials, due to strong Li ion conductivity, great compacted density (4.2 g cm^3^), reliability, heightened energy density and extended life span. Even after 30 years of its invention as LIB cathode, it is still the governing cathode material [22]**.** LiCoO_2_-based cathode materials in LiBs will demonstrate promisingly higher volumetric energy density efficiencies, which make them the most reliable materials for energy storage systems in commercial portable devices [23].

The research and development of electrode materials should be directed with a focus on exploring the internal materials of batteries, the formula of electrolytes, and the synthesis of new electrode materials [24]. In battery-related research, the design of charging strategy has been developed in the field of power electronics [25]**,** with a focus on charging strategies and circuit design. Although the sinusoidal charging strategy is being considered beneficial for suppressing battery aging, there are sporadic literature studies that are predominantly dedicated to exploring the impacts of charging methods on the changes in the internal materials of batteries after charging-discharging cycles as far as the knowledge of the authors is concerned. Therefore, this current research investigation is considered a novel attempt, where two efficient charging strategies (CC-Sinusoidal and CC-CV) are being compared and their influences on the evolutions in materials like graphite anode and LiCoO_2_ cathode after charging-discharging cycles have been reconnoitered and described. The research outcomes have highlighted the benefits of the CC-Sinusoidal charging method for extended battery cycle life. They will also provide a perspective on the impact of charging methods on the atomic level of electrode materials in the field of power electronics.

## 2 Materials and Methodology

### 2.1 Experimental process

**Fig. 1:**
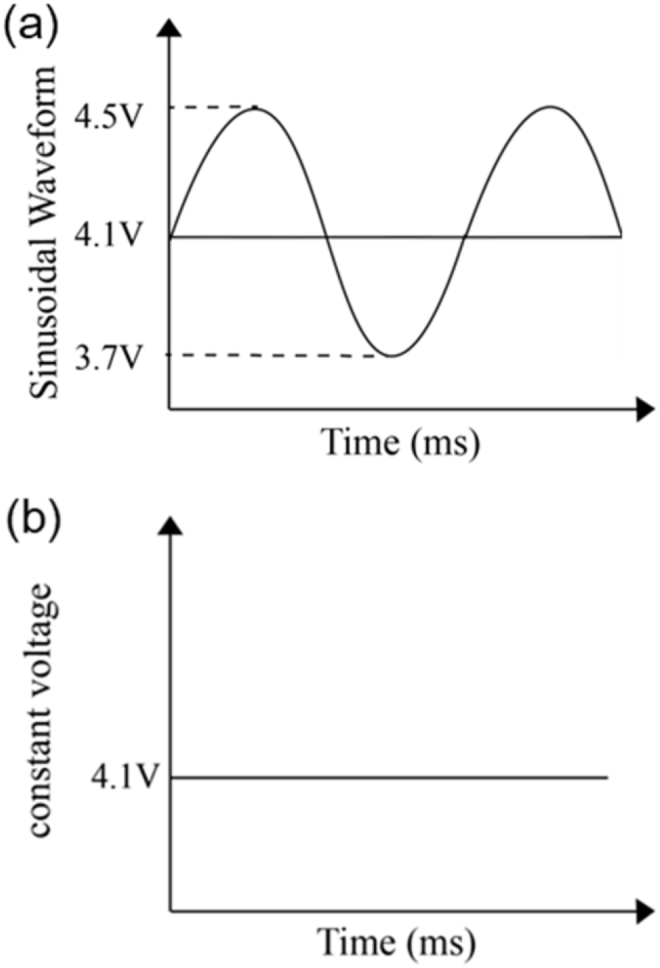
(a) Charging voltage for sinusoidal wave and for (b) constant voltage.

The research experiment used a DC power analyzer (N6705C, Keysight Technologies, Inc., Santa Rosa, CA, USA) to charge and discharge the battery. The charging methods were CC-CV charging and CC-Sinusoidal charging, while the discharge methods were both CC discharge. In order to fulfil the current efficiency requirements for battery charging, a fast-charging method was used to charge the battery at 3C. At the beginning, the battery was charged to a voltage of 4.1 V using CC charging method, and then it was converted to CV charging or sinusoidal wave charging, in order to maintain battery charging until the charging current reached 0.0021 A (0.02C). After the battery was fully charged, there was a cessation for 15 min, and then discharging was performed at a constant current of 1 C–3 V and again there was a 15 min pause. This was the charging-discharging cycle phenomenon followed for the Li battery. To prevent overcharging of the battery, a limit voltage of 5.0 V was set. The difference between the two charging methods was calculated on the basis of the following mechanism: After the completion of CC charging, the sinusoidal wave was a waveform with a voltage of 4.1 V as the reference, a top chord wave of 4.5 V, and a bottom chord wave of 3.7 V, and was charged at a frequency of 7000 s^−1^ until the battery was completely charged. This is clearly shown in Fig. 1(a). The CV charging law maintained 4.1 V until the battery was entirely charged, as shown in Fig. 1(b).

This study used a commercially available soft-pack lithium-ion battery (BYD Company Limited, Shenzhen, China) as the experimental material, with a capacitance of 105 mAh, limiting and saturation voltages were 5.0 V and 4.1 V, respectively. Two different charging methods like the CC-CV and the CC-Sinusoidal were applied and the detailed experimental process was as follows.

1. A charging-discharging cycle was performed for the batteries and a thermocouple thermometer was installed to monitor the temperature and room temperature of the upper and lower sides of the battery during the cycle. Three identical battery cells were selected for the charging-discharging under 100, 300, and 500 cycles.
2. To determine the impedance changes and aging degree of the battery under different cycles, a 20-h cessation was done every 25 charging and discharging cycles, and constant potential and current meter were used to measure the impedance of the battery. An effective circuit model after obtaining the electrochemical impedance spectrum was established
3. After the completion of the required charging-discharging cycles, the battery was placed in a glove box for disassembling. The electrode was removed first, and the surface was cleaned with dimethyl carbonate (DMC) solvent to remove residual lithium ions and impurities and it was then soaked in the solvent for 24 h and later placed in a vacuum oven for drying at a temperature of 40 °C.
4. Detection of the SEI film on the electrode surface and the degree of electrode damage was done with instruments like SEM, XRD, and Raman spectroscopy and then the electrode differences and impedance changes under different charging methods and charging times were compared.

### 2.2 Measurements with potentiostat/galvanostat

The potentiostat/galvanostat instrument (VersaSTAT 3, AMETEK Scientific Instruments) was used to measure the battery impedance under the constant current mode. Under accurately adjusted applied AC current with parameters set from the starting frequency of 150000 Hz to ending frequency of 0.01 Hz, and applied amplitude of 10000 μA RMS, the voltage and state of charge of the battery might be unaffected. The voltage ranges of the instrument parameters were ±6 V. The electrochemical impedance spectrum composed of the real and imaginary impedance parts of the battery measured by the instrument that can be imported into the software to analyze the data and obtain an equivalent circuit model composed of electronic components. After the equivalent circuit model was established, data from various electronic components will be generated for analysis and by paralleling the data, the trend of the internal impedance value of the battery can be obtained.

### 2.3 Emission scanning electron microscope measurement

A field emission scanning electron microscope (FE-SEM, ZEISS-Sigma, Carl Zeiss AG, Jena, Germany) was used to visualize the aged electrode surface, where the electrodes were stuck with copper adhesive on the carrier platform and placed on the sample rack for SEM detection. The electrode material itself contained carbon elements with conductivity, which could avoid overcharging the surface. This may result in extremely high brightness and poor image quality and gold or platinum coating on the sample surface was not required. The accelerating voltage of the parameter was set to 10 kV and the detector was an In lens for clearly observing the sample for surface contamination.

### 2.4 X-ray diffractometer measurement

In order to understand the crystal size variation of the electrode materials caused by different charging methods and cycles under fast charging, an X-ray diffractometer (XRD, PANalytical - X'Pert ³ Powder, Malvern Panalytical Ltd., Malvern, UK) with a target material of metallic copper was used. The two electrode samples were cut and placed on a glass slide and inserted into an XRD platform for measurement. The X-ray source was a rotating anode copper target (Cu Kαλ = 0.15418 nm), a voltage of 45 kV and a current of 40 mA, and the working range of the goniometer system was (2θ) Set at 10°–90°, with a step size of 0.0131303°. The spectrum produced by XRD measurement was confirmed through database or literature comparison to determine the atomic types and positions of the internal composition of the sample. The intensity of the spectrum was used to compare the differences in samples under different experimental parameters. Data analysis software was used to analyze the full width at half maximum (FWHM) and crystallization peak area, and then brought into the Scherer Equation (1) and the crystallinity index (CI) formula, as shown in Equation (2), grain size and related sample crystal structure information can be obtained.

(1) $$\text{Crystallite}\;\text{size}\;K\left(D\right)=\frac{K\lambda }{\beta \;\cos\;\theta }$$

(2) $$\text{Crystallinity}\;\text{index}\;\left(\text{CI}\right)=\frac{\text{area}\;\text{of}\;\text{crystalline}\;\text{peaks}\;}{\text{area}\;\text{of}\;\text{all}\;\text{peaks}}\times 100$$

Where K in Equation (1) is the dimensionless form factor. β is the half-width of the diffraction peak of the sample which is usually 0.89. λ is the X-ray wavelength (Cu Kαλ = 1.5418 Å).

### 2.5 Raman spectrum measurement

In order to determine the degree of damage to electrode materials and compare the differences under different cycles and two charging methods, Raman spectrometer (RAMaker, ProTrusTech Co. Ltd., Tainan, Taiwan) was selected, and a laser wavelength of 532 nm was used. The other parameters were laser intensity of 50 %, exposure time of 60 s, and accumulation times of 5.

## 3 Results and discussion

The experimental process and method designed in Section 2 were used to obtain instrument measurement data. 100, 300, and 500 charge-discharge cycles were set as fixed variables, and the experimental results of battery capacitance, charge-discharge cycle temperature, battery impedance, SEM spectra of electrodes, XRD spectra of electrodes, and Raman spectra of electrodes were presented based on the results of CC-CV charging and CC-Sinusoidal charging.

### 3.1 Battery capacity and charge-discharge cycles

**Fig. 2:**
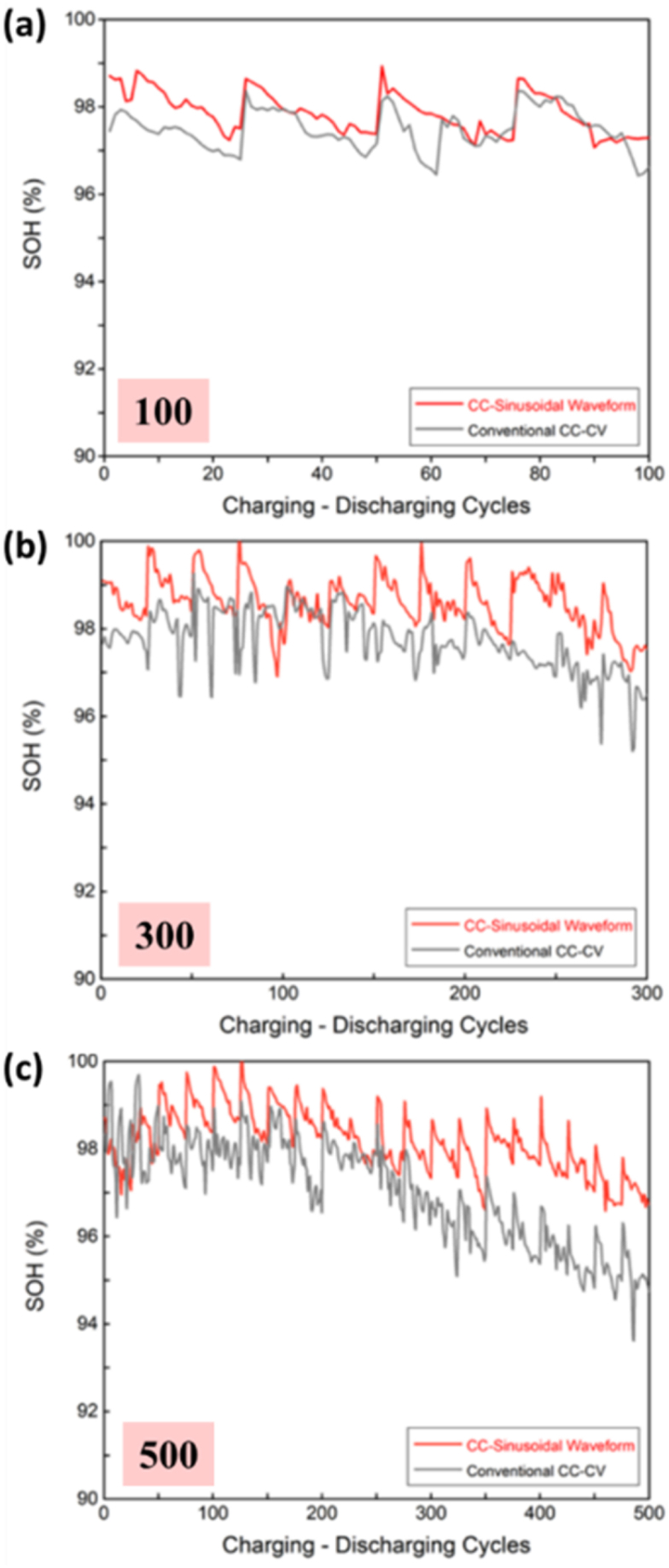
Capacity of (a) 100, (b) 300, and (c) 500 charge-discharge cycles.

The comparison of the SOH of the battery under two different charging modules based on the number of cycles, revealed that the SOH curves for both the modules were roughly the same after 100 cycles, with SOH values of 96.62 % and 97.29 %, respectively. This indicated that there were no significant differences from the rated capacitance of the original battery and battery aging phenomenon had not occurred. When the battery was charged and discharged for 300 cycles, the SOH curve using the CC-CV charging method slightly decreased to 96.49 %, while the SOH of the sinusoidal module remained unchanged at 97.54 %. The SOH curve of both charging methods showed a downward trend while the number of battery charging and discharging cycles was continuously increased to 500, especially for the CC-CV charging method (Fig. 2). After cycling 300 times, the SOH began to significantly decrease to 94.75 % and although the battery was not severely damaged, increasing the number of cycles might cause a sudden downfall in the capacitance. Meanwhile, the CC-Sinusoidal charging method started with a slight decrease in capacitance to 96.73 % after approximately 400 cycles, which was higher than that of the CC-CV charging method. A significant previous research study from our team was performed to suppress the anode electrode degradation in Li batteries by sinusoidal charging method [26]**.** The batteries were charged for 600 charge discharge cycles and the capacitance measured in CC-CV was 80 %, whereas that of sinusoidal was well ahead with 94 %, thus revealing that it was the advantageous method.

The temperature change during the discharge process of the battery under both the charging methods was higher than the charging process, whereas the temperature variations throughout the charging process of both the charging methods were approximately similar with the charging-discharging cycles. However, as the number of charging-discharging cycles increased to 500, the temperature during the charging process exceeded 30.5 °C, indicating an increase in the resistance of lithium ions embedded in the graphite negative electrode. However, the charging-discharging cycles did not surpass 30.5 °C at 300 and 100 cycles and interestingly during the temperature change of the discharge process, the maximum temperature for both charging methods was approximately amid 31.5 °C and 32.0 °C under different charging and discharging cycles. **Li et al**. [27], have justified the results of this study by mentioning that the sinusoidal charging method had a great role in improving the charging period, charging efficiency, controlling the battery rising temperature and thereby having positive contributions to the overall lifespan of the battery.

### 3.2 Impedance of the battery

The impedance inside the battery was determined through AC impedance analysis, known as electrochemical impedance spectroscopy (EIS). Each reaction or process in EIS can be represented by a circuit composed of resistors, capacitors, inductors, or other electronic components with different phase differences (θ), and consequently, different Nyquist impedance spectra were generated [28,29]. For lithium-ion batteries equipped with graphite negative electrodes, the curve on the graph composed of an additional half circle related to SEI on the surface of the graphite electrode [30,31]. The impedance of the battery was measured after 25 cycles of charging and discharging followed by a 20-h interlude. The values of electronic components in the equivalent circuit model in Table 1 have been compiled. The first impedance (R_S_) is the ohmic impedance, followed by the parallel connection of the series capacitor (C_SEI_) and SEI film impedance (R_SEI_), followed by the analogous connection of the negative double-layer capacitor (C_DL1_) and charge transfer impedance (R_CT1_). Finally, the charge transfer impedance (R_CT2_) was linked in series with the diffusion impedance (W), which formed a parallel connection with the positive double-layer capacitor (C_DL2_). Several key points can be sorted out from Table 1 as follows:

**Table 1:** Numerical values of electronic components in equivalent circuit models.

Cycles	CC-CV						Sinusoidal wave					
	100		300		500		100		300		500	
	pristine	No. 100	Pristine	No. 300	pristine	No. 500	pristine	No. 100	pristine	No. 300	pristine	No. 500
R_S_	0.11	0.12	0.11	0.23	0.12	0.12	0.11	0.12	0.11	0.13	0.11	0.12
CPE_SEI_-T	9.96E-4	1.99E-3	9.54E-4	1.70E-3	1.18E-3	1.69E-3	1.09E-3	1.54E-3	1.31E-3	1.64E-3	1.38E-3	1.27E-3
R_SEI_	0.042	0.038	0.041	0.028	0.042	0.028	0.042	0.036	0.051	0.035	0.049	0.028
CPE_DL1_-T	0.022	0.029	0.021	0.037	0.023	0.035	0.026	0.035	0.022	0.036	0.021	0.037
R_CT1_	0.62	0.51	0.56	0.32	0.68	0.30	0.72	0.65	0.80	0.45	0.63	0.34
CPE_DL2_-T	0.080	0.081	0.078	0.079	0.081	0.079	0.085	0.075	0.082	0.071	0.080	0.078
R_CT2_	0.83	0.73	0.74	0.49	0.80	0.67	0.86	1.12	1.13	0.74	0.93	0.72
W_1_–R	0.53	0.60	0.65	0.67	1.69	0.63	0.63	2.64	2.61	3.46	0.57	0.73
Error(%)	4.65	3.98	6.44	7.62	7.43	5.21	5.22	6.45	5.53	5.87	4.63	7.85

Primarily, based on the impedance of the electrolyte, the difference between the battery afore and later to charging and discharging was observed to be insignificant, except for the substantial increase in impedance after 300 cycles of the CC-CV charging method, which might be due to the electrolyte quality of the battery.

Primarily, based on the impedance of the electrolyte, the difference between the battery afore and later to charging and discharging was observed to be insignificant, except for the substantial increase in impedance after 300 cycles of the CC-CV charging method, which might be due to the electrolyte quality of the battery. Analogous impedance rises in Li-batteries have been reported under CC-CV charging methods by **Ko and Chen,** [32] have revealed that this particular method accelerates electrolyte decomposition leading to impedance growth and battery calendar life reduction. Furthermore, from the perspective of CPE_SEI_-P, value nearing 0 indicated resistance and a decrease in impedance after charging-discharging cycles, which also designated that the battery performance was unaffected by SEI film formation. Then, there was a reduction in the impedance of other components like R_CT1_ and R_CT2_ after the charging-discharging cycles. It was inferred that the quality of the original battery sample might be deprived or the storage and discharging time may be lengthy. After charging and discharging, the effectiveness of battery activation was obvious and the cycle numbers was the foremost phenomenon that impacted the operation of lithium ions inside the entire battery. Lastly, the analysis data of the battery was in contrast with the Nyquist plot, but EIS measurement was greatly influenced by factors such as time, environment, instruments, and human factors, which might have triggered errors in data. Impedance increases by **Sangeetha et al**. [26], are in accordance with this study, where during the 100th cycle, under CC-CV charging and room temperature, the impedance of every part of the Li-battery increased. By 400th cycle, the CC-CV method had impacted a 0.94Ω of impedance compared to only 0.08 Ω by the sinusoidal charging. These above mentioned justifications have proved that the outcomes of the experiment are acceptable.

### 3.3 SEM spectrum of electrodes

According to the 50K magnification image displayed in the supporting information (SI) Fig. S1, it can be observed that the original positive electrode was not covered by a thin film, which was formed by strip aggregation compared to other charged electrodes, which might be the cathode electrolyte interface layer. The FE-SEM observed the surface microstructure of the negative electrode, after 600 cycles, where absence of SEI films was observed with sinusoidal charging method [33]**.** On contrary the CC-CV charged electrodes had SEI films on their surface. They had substantiated the appearance by declaring that the CC-CV method diminished the electrode quality. Correspondingly, in this present study, after 100, 300, and 500 cycles of charging and discharging, the positive electrode exhibited diminutive differences from the SEM image. In addition, as publicized in the 50K magnification illustration in Fig. S2, there is a thin film covering several layers. After 100, 300, and 500 cycles of charging and discharging, the positive electrode was almost identical to the SEM image. However, compared with the electrode of the CC-CV charging method, the difference was considered to be inconsequential. Therefore, it was speculated that the lithium cobalt oxide positive electrode material may have a stable structure and noteworthy damages did not occur under charging methods and cycles.

**Fig. 3:**
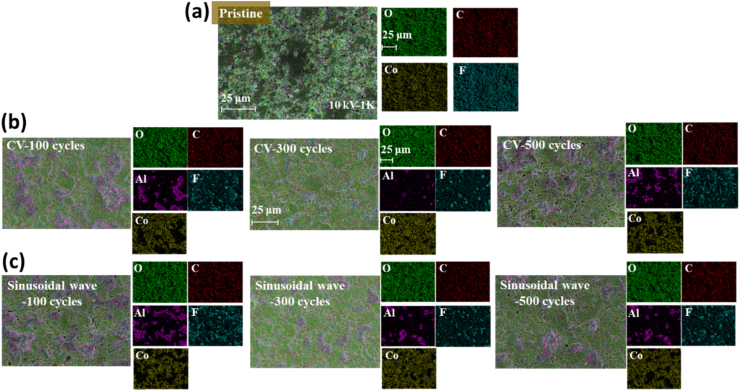
Distribution diagram of positive electrode EDS elements (a) pristine (b) CC-CV charging with different cycles and (c) CC-Sinusoidal charging with different cycles.

After measuring EDS, the distribution of each element at a magnification of 1K is was obtained as shown in Fig. 3 and the element percentage in the materials has been displayed in Table 2. The presented elements included the ones that were the constituents of the lithium cobalt oxide positive electrode, and the F element derived from the electrolyte. In addition, it was also inferred that Al element was absent in the electrodes that were not subjected to charging-discharging cycles, while it was present in other electrodes after several cycles of charging. Nevertheless, this justified the phenomenon that the metal Al collector might have been damaged after multiple cycles of charging-discharging and persisted on the electrode surface. According to Table 2, the proportion of C, O, and Co elements was similar under different charging-discharging cycles and among them, the atomic amount percentage of F element decreased significantly from 17.5 % to 10 % after several charging-discharging cycles. Parallel research work by **Xiong et al** [34], have exhibited that in-depth analysis of the SEI membrane revealed the presence of components like lithium (Li 1s), oxygen (O 1s), carbon (C 1s). They concluded that the sinusoidal charging method was proficient and effective in mitigating the progress of SEI membrane in the electrodes.

**Table 2:** Element percentage of positive electrode.

CC-CV charging					
	C (At%)	O (At%)	Co (At%)	F (At%)	Al (At%)
Pristine	35.9	31.0	15.5	17.5	0.0
100 cycles	37.2	33.2	14.0	10.4	5.2
300 cycles	37.9	36.3	18.3	6.7	0.8
500 cycles	39.0	31.6	14.4	10.1	4.8
CC-Sinusoidal charging					
	C (At%)	O (At%)	Co (At%)	F (At%)	Al (At%)
Pristine	35.9	31.0	15.5	17.5	0.0
100 cycles	37.3	31.7	12.7	12.0	6.4
300 cycles	37.5	35.2	18.0	7.4	1.9
500 cycles	38.4	33.9	15.5	8.8	3.4

Consequently, it was construed that F element had gradually combined with lithium ions to form LiF during the charging and discharging process, which was deposited on the surface of the negative electrode as one of the components of the SEI film. After measuring EDS, the distribution of each element at a magnification of 1K as displayed in Fig. 3(b and c) was acquired and it not only included the original lithium cobalt oxide positive electrode element but also F element obtained from the electrolyte. Moreover, the atomic amount percentage of each element in the material was determined as displayed in Table 2. In addition, compared to Fig. 3(a), Al element was prevalent, regardless of the charging method used and after several cycles of charging and discharging. From Tables 2 and it can be observed that the proportion of C, O, and Co elements was similar under various charging and discharging cycles, but the atomic amount percentage of F element decreased from 5 % to 9 % from its original atomic number percentage of 17.5 %, after several charging-discharging cycles.

Therefore, it was speculated that the F element gradually combined with lithium ions during the charging and discharging process to form lithium fluoride, which was deposited on the surface of the negative electrode as one of the components of the SEI film. If two different charging methods would have been compared and if the results measured by EDS might have been the same, it could have been deduced that the positive electrode material was less prone to significant changes due to the number of charging-discharging cycles and charging methods.

From Fig. S3(a) and Fig. S3(b), it was observed that the original negative electrode surface was smoother and had a thin film layer covering the graphite surface called the SEI film. When the number of charging-discharging cycles increased, the SEI film on the negative electrode surface became significantly thicker, especially after 500 cycles, as exhibited in Figs. S3(g) and Fig. S3(h), and the thickness distribution was uneven. Therefore, it could be confirmed that the SEI film gets thicker as the number of cycles increased, which is a phenomenon that results in battery aging and battery lifespan decrease. According to Fig. S4(a), the SEI film exhibited thickening signs under different charging and discharging cycles, when the sine wave charging method was used, but the difference was slighter compared to the original negative electrode surface as shown in Fig. S4(b). By comparing the results of two charging modules, it was comprehended that when the battery was charged and discharged for 500 cycles, the SEI film produced by the CC-CV method was significantly thicker than the one produced by the CC sine wave charging method. Consequently, it was also confirmed that the CC sine wave charging method was capable of reversing the precursor reaction of the SEI film, reducing the generation of SEI film, and thereby causing an overall plummeting in battery aging.

**Fig. 4:**
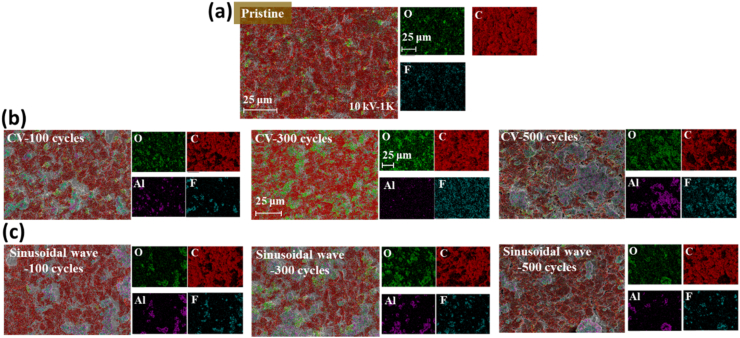
Negative electrode EDS element distribution diagram (a) pristine, (b) CC-CV charging with different cycle times, and (c) CC-Sinusoidal charging with different cycle times.

Quantitative and qualitative analysis of the elements in the negative electrode were obtained by the EDS measurement and the scanning is performed at a magnification of 1K, and two different charging methods have been discussed and compared as follows. After measuring EDS, the distribution of each element in the 1K graph as shown in Fig. 4 and the atomic amount percentage of each element in the material as presented in Table 3 were obtained. Fig. 4(a) shows the element C of the original negative electrode and the element F obtained from the electrolyte. After the CC-CV charging-discharging cycle, as shown in Fig. 4(b), the O element formed the SEI film, which was converted to lithium oxide or lithium carbonate and accumulated on its surface. **Du et al** [35]**,** have reported an identical outcome in their study where CC-CV charging produced higher degrees of lithium fluoride, lithium oxide and lithium carbonate compared to that of sinusoidal charging. Such reduced amounts of Li in sinusoidal method, had less bond energy and made the charge discharge cycles much easier and meritoriously resulted in protracted battery service life. In addition, Al element also appeared after 100 cycles, indicating a defect in the metal aluminum collector. Table 3 demonstrated the fact that as the number of charging and discharging cycles in the battery increased, the atomic number percentage of element C decreased from 90.3 % of the original battery to 71.7 % at 500 charging and discharging cycles, while the weight percentage of element F and O escalated, and the atomic number percentage of element O was about 10 % higher than the original battery. It can be inferred from the compounds such as lithium carbonate, lithium fluoride, and lithium oxide composed of the SEI film described in the first chapter that these elements formed a SEI film on the negative electrode surface and gradually thickened with increasing charging and discharging cycles.

**Table 3:** Element percentage of negative electrode.

CC-Sinusoidal charging				
	C (At%)	O (At%)	F (At%)	Al (At%)
Pristine	90.3	7.7	2.0	0
100 cycles	87.1	8.6	2.9	1.5
300 cycles	87.3	9.5	2.2	1.0
500 cycles	85.8	10.6	2.7	1.0
CC-CV charging				
Pristine	90.3	7.7	2.0	0
100 cycles	83.2	12.8	3.3	0.7
300 cycles	80.9	14.0	4.7	0.4
500 cycles	71.7	17.6	8.1	2.6

**Fig. 5:**
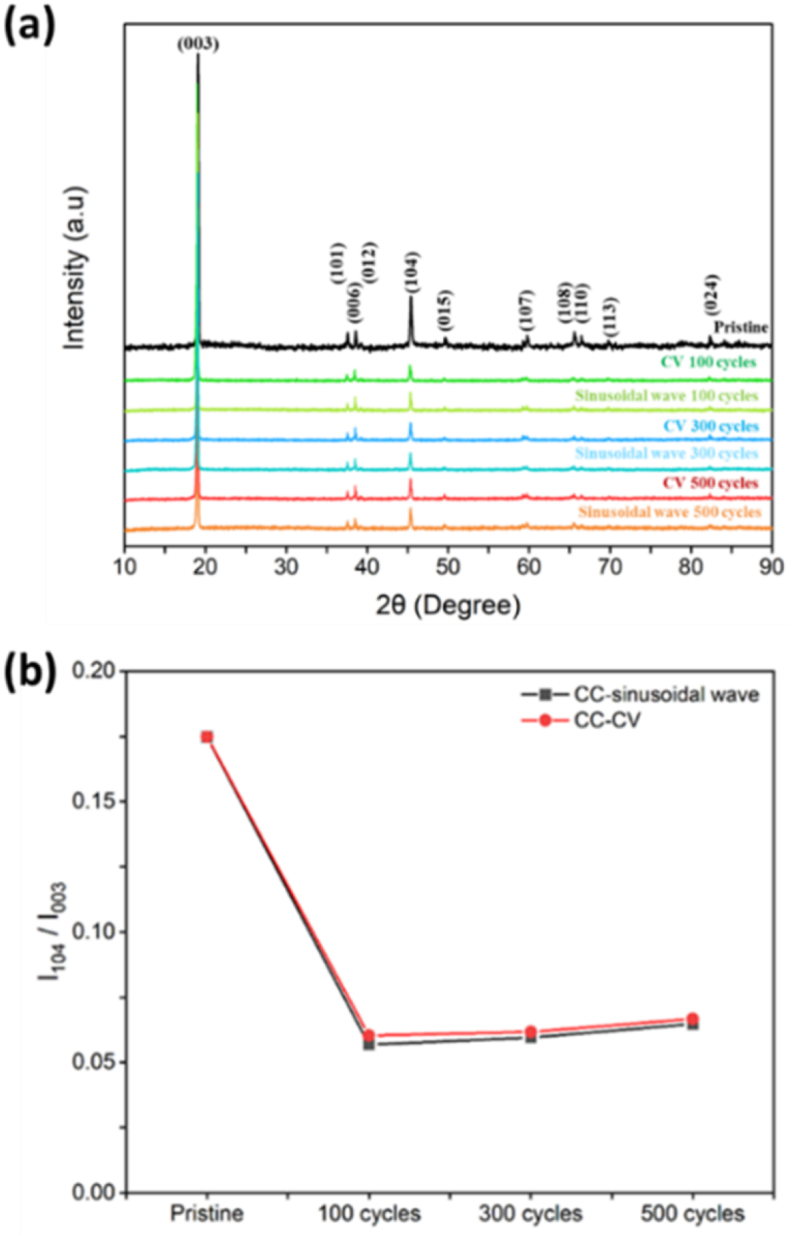
(a) XRD spectra of different charging and discharging cycles of the positive electrode under two different charging modules. (b) The number of cycles between the two charging methods affects the I_104_/I_003_ variation of the positive electrode.

The distribution of various elements in the electrode is exposed in Fig. 4(c) and the atomic amount percentage of each element in the material is presented in Table 3 based on the results of EDS measurement of the 1K SEM image after the charge-discharge cycle of the CC-sinusoidal method. Compared with the element distribution diagram in Fig. 5(a) and (c), except for the addition of Al element to the original electrode, the element distribution diagrams under the other two charging methods were similar.

From Tables 3 and it is obvious that as the number of charges and discharges increased, the atomic number percentage of element O gradually upsurged in electrodes using the sine wave charging method, but the difference in other elements was insignificant, indicating that this method can inhibit the growth rate of SEI films. Compared to the CC-CV charging method, the percentage of elemental atoms in the main growth of SEI films was relatively less, which corresponded to the SEI diagram of the negative electrode surface. Therefore, it was preliminarily determined that using the CC-Sinusoidal charging method would have a lower impact on battery aging compared to the CC-CV charging method.

### 3.4 XRD pattern of electrodes

Layered lithium cobalt oxide (LiCoO_2_) was used as the positive electrode material for batteries in this study and was characterized through XRD technique. As a result, a spectrum of intensity and double scattering angle were obtained. From this spectrum, the characteristic peak of LiCoO_2_ was obtained and the differences between literature and experiments were compared. The position of the double scattering angle was the same, indicating that the experimental material was correct. At that time, based on the XRD patterns, Scherer equation and crystallinity index were used to calculate the crystal cell parameters of the sample. LiCoO_2_ materials had two crystalline phases, one was a layered structure and the other one, a spinel-type structure. The XRD patterns of the two were analogous, making it difficult to distinguish between them and as a result, the ratio of the lengths of the a-axis and c-axis were utilized as a judgment indicator. The c/a ratio of about 4.90 is a spinel structure, while about 5.00 is a layered structure. The characteristic peaks (003 and 110) in the spectrum represented the stacking direction and width of the CoO_2_ layer, respectively, and the crystal size of the product was calculated [36,37].

In Fig. 5(a), the Miller indices of the XRD characteristic peaks of LiCoO_2_ cathode material at 19.09°, 38.58°, and 45.41° are (003), (006), and (104), respectively. The stronger the characteristic peak of (104), the more the material structure proliferated in this arrangement direction. After several cycles of charging and discharging, the increase in peak intensity (104) indicated a change in the arrangement of crystal lattice planes in the material. The characteristic peak with a lattice surface of (003) represented the layered structure of lithium cobalt oxide material, while the characteristic peak with a lattice surface of (104) signified the basic unit of the layered structure (Co–*O*–Co). Therefore, the interlayer spacing (d) between materials was calculated using Bragg's law, where the wavelength (λ) was 1.5418 Å and the diffraction multiple (n) was 1. The angle between the incident wave and the scattering plane (θ) was adopted. The characteristic peak position can be determined by a factor of half, and it can be incorporated into Equation (1) to obtain the inter planar spacing of each lattice surface, as shown in Table S1. The position of the characteristic peak on the graph did not shift with different charging methods and cycles, and there was no significant difference in crystal plane spacing, indicating that the structure of this positive electrode material was stable and did not undergo prominent damage. According to Bravais lattice theory, the structure of LiCoO_2_ was a hexagonal lattice and therefore, the Miller index and the crystal plane spacing obtained above was substituted into the calculation to obtain the lattice constants a and c. Due to the relationship between the hexagonal crystal system a = b, a value close to 5 from c/a indicated that the LiCoO_2_ electrode was a layered structure without a spinel structure.

In addition, the c/a value could also be used to determine the interlayer spacing size of the material and the electrodes in this study were disassembled after discharging the battery. According to Table S2, the c/a value of the original positive electrode was 4.97a and after it was subjected to 100 cycles of both the types of charging and discharging strategies, this value increased, thus indicating that charging and discharging had an activation effect on the LiCoO_2_ electrode structure. After 300 or 500 subsequent charging and discharging cycles, the c/a value reduced, demonstrating that lithium ions did not entirely return to the positive electrode during discharge, leading to an escalation in structural disorder and a diminution in the c-axis. It was also ventured that lithium ions might have experienced losses in the negative electrode or electrolyte. The reason may be that during the charging process of the battery, lithium ions detached from the positive electrode structure of LiCoO_2_ and the cobalt ions transformed from positive trivalent to positive tetravalent, resulting in a phase change in the structure. This occasioned in an increase in disordered structure and a decrease in ordered structure, while discharge was the completely contradictory. By comparing the two types of charging modules, it was incurred that the c/a value was the lowest when the CC-CV charging was 500 cycles. Paralleling the strength of the main characteristic peaks (003) and (104) of LiCoO_2_ materials, it was found that their ratio represented the performance of lithium cobalt oxide. The higher the strength ratio, the more content the crystallization sequence was in the 104 direction. Fig. 5(b) confirmed that the I_104_/I_003_ ratio of the original electrode was 0.1748, which significantly decreased to about 0.05–0.06 after charging and discharging cycles, indicating an increase in the integrity of the crystalline material and a neat sorting. The more charging and discharging cycles there were, the higher was the I_104_/I_003_ ratio, which corresponded to the above c/a value. According to the literature, the main characteristic peaks of lithium cobalt oxide positive electrode were at 003 and at 104. Therefore, the FWHM of these two characteristic peaks was achieved using analysis software and were incorporated into Equation (2), with a shape factor of 0.9 and an X-ray wavelength of 1.5418 Å, β The half-width of the diffraction peak of the sample was calculated to obtain the grain size as shown in Table S3.

**Fig. 6:**
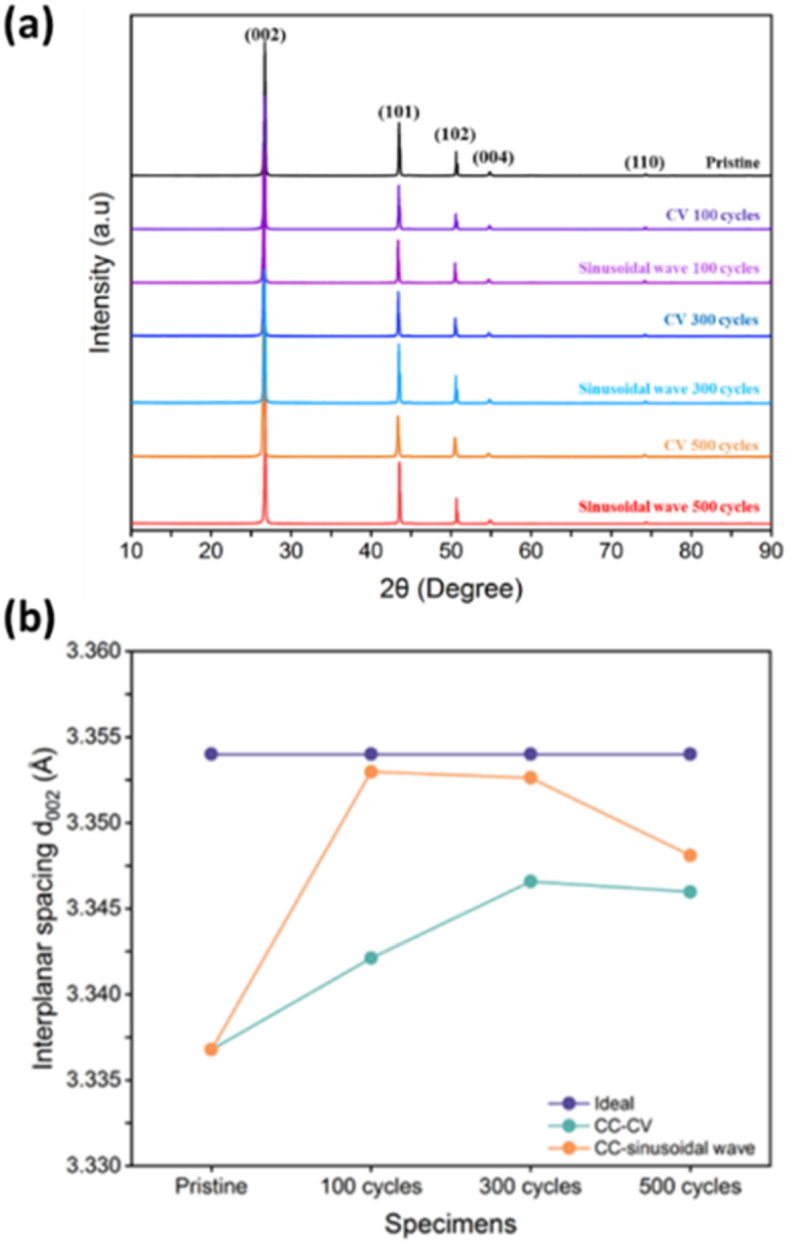
(a) XRD spectra of different charging and discharging cycles of the negative electrode under two different charging modules (b) Comparison diagram between the ideal interlayer spacing (d_002_) of graphite electrodes and the actual interlayer spacing (d_002_).

The characteristic peak positions of the graphite negative electrode were approximately 26.72°, 43.51°, 50.64°, 54.82°, and 74.29°, with Miller indices of (002), (101), (102), (004), and (110), as publicized in Fig. 6(a). The diffraction peak (102) suggested that a small amount of carbon black material may be doped into the reflecting surface to increase electrode conductivity. Therefore, according to Fig. 6(a), it can be interpreted that the diffraction peak intensity under CC-CV charging was lesser than that of the original electrode, and the diffraction peak intensity under CC-Sinusoidal charging, which was speculated to be caused by consumption during CC-CV charging and discharging. The other peak surfaces (002), (101), (004), and (110) were diffraction peaks of graphite, with (101) diffraction peak using Cu Kα The reflection peak caused by the target material, as presented in Fig. 6(a), signposted that the diffraction peaks (004) and (110) remained almost unaffected. To better understand the characterization of graphite electrodes, Equation (1) was used to calculate the interlayer spacing (d_00ι_) as indicated further; The wavelength (λ) of 1.5418 Å Diffraction peak (00ι) and diffraction multiple (n) of 1 and half times position substitution, where ι equal was to 2 or 4, the interlayer spacing was calculated. The d_002_ of ideal graphite was 3.354 Å. From Table S4, it can be observed that the slight increase in interlayer spacing of the original graphite electrode after cyclic charging and discharging indicated that lithium ions had instigated to embed into the negative electrode, and the interlayer spacing was closer to the ideal electrode. Perhaps, it might have affected the electrode activation after charging and discharging cycles. Therefore, the d_002_ of the original negative electrode was 3.337 Å. After two charging and discharging cycles, the d_002_ of the negative electrode was approximately 3.340 Å to 3.350 Å. In Fig. 6(b), it can be clearly observed that after two types of charging and discharging, the d_002_ layer spacing of the original electrode approached the ideal value, especially in the sine wave charging method. However, as the number of charges and discharges increased, the distance between the electrode d_002_ layers under both charging methods deviated from the ideal value, resulting in a more severe depart from the CC-CV charging method. Erstwhile study from our team [38]**,** have determined through Lorentz fitting intensity ratios that the 2D-band concomitant with graphitic layers of the electrode was adjacently interconnected to the intercalation of Li ion them and this made the fact clear that the sinusoidal charging method had a great role to play in subduing this interjection than the other CC-CV technique.

According to the previous research investigations [[39], [40], [41]], the main characteristic peaks of graphite negative electrode were (002) and (004). Therefore, the half width at half height of these two characteristic peaks was obtained using analysis software and substituted into Equation (2). Its shape factor was 0.9, and the X-ray wavelength was 1.5418 Å, β The half-width of the diffraction peak of the sample can be calculated to obtain the grain size (L_c_) as shown in Table S5. It was found that the size of graphite grains decreases with the increasing number of electrode cycling charges and discharges, especially after using the constant current constant voltage charging method for 500 cycles. The grain size of the diffraction peak (002) decreased from 45.884 nm to 38.605 nm in the original electrode, while the grain size of the diffraction peak (004) decreased from 32.748 nm to 29.725 nm in the original electrode. It is inferred that after 500 cycles of charging and discharging, the structure of the graphite electrode was somewhat peeled off, resulting in grain size reduction. On the contrary, in the CC-Sinusoidal charging method, although the grain size of the two diffraction peaks slightly decreased after charging and discharging cycles, the trivial decrease indicated that the impact on the graphite electrode was relatively insignificant after multiple charging and discharging of the battery. The discussions mentioned have well substantiated the results of the current study.

### 3.5 Raman spectra of the electrodes

Raman spectroscopy technology was used in this study to identify chemical bonds in molecules and solids, doping in semiconductor materials, and even different forms of carbon materials. The Raman spectra of LiCoO_2_ confirmed that greater the number of charge-discharge cycles, lesser was the peak intensity and especially after 400 cycles, it almost disappeared [42,43]**.** Graphite possessed symmetry and small dipole characters and Raman spectra using light scattering as a detection principle could exhibit high-intensity peaks. The main characteristic peak of single-layer graphene in the spectrum was in the G band of 1582 cm^−1^, which was related to the double degenerate phonons in the Brillouin zone center. This was the only band with a first-order Raman scattering process, which represented the vibrational mode of carbon as sp^2^ and reflected structural integrity. Once graphene is stacked as a disordered structure or with structural edge damage, it will exhibit a D band at 1350 cm^−1^ on the spectrum, which involves inelastic scattering of one phonon and one defect, known as the second-order Raman scattering process. The frequency of the G′ band is approximately twice that of the D band. **Chen et al.,** [44] have displayed their Raman spectroscopy results to have a clear understanding about the various impacts of charging methods on the graphite electrodes. The spectra consisted of D-, G-, and 2D-bands. The intensities of the Raman spectra and their ratios were investigated to reveal the actuality that the CC-CV method had deteriorating effects on the graphite electrodes, compared to sinusoidal. Hardwick et al. [45]**,** refers to the G′ band as a 2D band and a weak small peak with a D′ band was observed on the spectrum at approximately 1620 cm^−1^, indicating the characteristic of disorderly induction The G, G′, and D bands mentioned above are crucial for analyzing the Raman spectrum of graphite. The higher the intensity ratio (I_D_/I_G_) between the defect (D) band and G band, the more defects there are in the graphite electrode structure; The smaller the strength ratio (I_2D_/I_G_) between the G′ band and G band, the more graphene layers there are, the more severe the stripping of the electrode material.

**Fig. 7:**
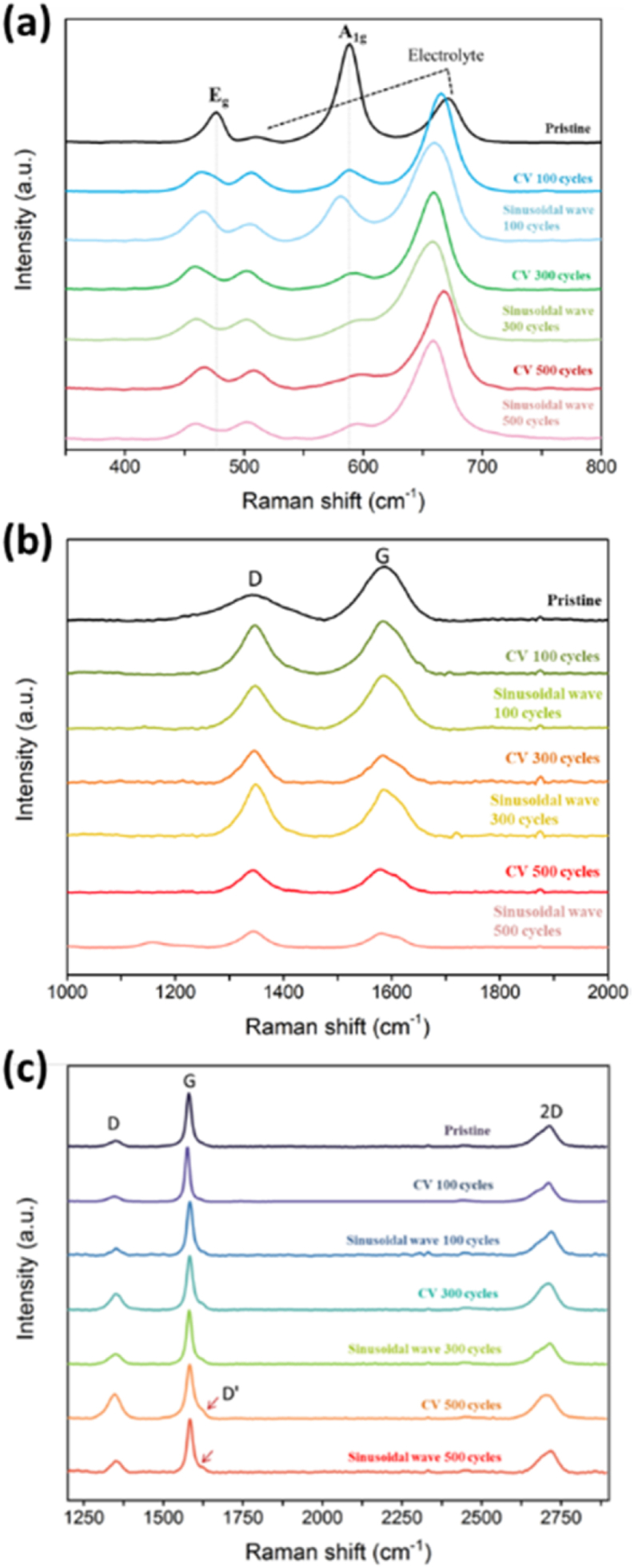
(a) Raman spectrum of positive electrode LiCoO_2_. (b) Raman spectrum of graphite negative electrode. (c) Raman spectra of graphite negative electrode with two different charging methods and different charging cycles.

In reference to the data obtained from the lattice vibration of LiCoO_2_ measured by Raman spectroscopy, literatures [46,47] specified that there were two Raman characteristic peaks of lithium cobalt oxide called A_1g_ and E_g_ at Raman shifts of about 589 cm^−1^ and 477 cm^-^1, respectively. The A_1g_ mode is the stretching mode of oxygen atoms parallel to the c-axis vibration, i.e. Co–O; The E_g_ mode is the bending mode of oxygen vibrating along the a and b planes, i.e. O–Co–O. Other characteristic peaks were caused by residual electrolytes, as shown in Fig. 7(a). The peak intensity of A_1g_ and E_g_ modes decreased significantly with the increase of charging-discharging cycles. It was also speculated from previous SEM and XRD that the positive electrode structure will not be severely damaged and the characteristic peak value may decrease due to the strong electrolyte signal compared to the previous one. As described in the literature in Section 2, the cathode material deteriorated, and when laser light was irradiated on lithium cobalt oxide material, the transmittance decreased, causing light reflection and increased the absorption coefficient of light.

Graphite or carbon black material is usually added to the positive electrode material to increase conductivity. After characterization of the carbon material C–C by Raman spectroscopy, it was found that there was no characteristic peak (D′) of graphite on the spectrum, and it was determined that the additive was carbon black material. Literature evaluation [40]**,** suggested that the main characteristic peaks of carbon black were the G band and the D band, located on 1347 cm^−1^ and 1587 cm^−1^ of the spectrogram, respectively. The G band represented an ordered arrangement of the structure, while the D band, a disordered arrangement of the structure. In Fig. 7(b), the intensity of the G band decreased with increase of charging and discharging times and it widened. However, the intensity of the D band gradually increased after 300 charging-discharging cycles and a higher intensity in the D band than in G band indicated an increase in defects or disordered structures in the carbon material.

The negative electrode plane was cut and placed on the Raman spectrometer platform, with a measurement range set between 1200 cm^−1^ and 2900 cm^−1^. Three peaks of graphite structure were generated between 1000 cm^−1^ and 3000 cm^−1^, namely defect peak (D) at 1350 cm^−1^, monolayer graphene characteristic peak (G) at 1582 cm^−1^, and second-order disordered peak (2D). After data comparison, the D, G, and 2D bands on the spectrum were 1350.2, 1580.2, and 2713.2 cm^−1^, respectively (Fig. 7(c). Interestingly, it was noted that more the charging and discharging cycles, more G peaks represented the integrity of the graphite structure. They gradually appeared together, with a D′ small peak at approximately 1622 cm^−1^ representing disorderly induction. Especially, 500 cycles under CC-CV charging method were the most obvious. As the number of cycles augmented, the D peak of the graphite electrode under CC-CV charging method became greater, and the D peak intensity was significantly higher than that of the graphite electrode under the other charging method, thereby indicating that higher cycles will cause graphene stacking disorder or damage of structural edge of graphite electrode. The higher the intensity of the 2D peak (G′) on the spectrogram, the more defect-free graphene was stacked in the y-axis direction. After two different charging and discharging methods and different numbers of the cycles, the difference in 2D band was insignificant, specifying that the two charging methods and 500 charging-discharging cycles had diminutive effects on the graphite electrode [43,48].

### 3.6 Noteworthy Deliberations

From the SEM images, it was ventured that less differences occurred in the positive electrode between lithium cobalt electrode and graphite electrode batteries. This may be due to the stable structure of lithium cobalt oxide positive electrode material, which was unaffected by charging methods and cycle numbers. Nonetheless, according to EDS, the F element gradually combined with lithium ions during the charging and discharging process to form lithium fluoride, which was deposited on the surface of the negative electrode as the major SEI film component. The SEI film appeared clearly on the negative electrode and is visible in the SEM with 500 cycles of CC-CV, and verified by the atomic percentage in EDS. The positive electrode of XRD had an activation effect after less cycles of charging and discharging, and the electrode structure was well-ordered. But as the number of cycles increased, after 300 or 500 charge-discharge cycles, the c/a value decreased, indicating that lithium ions did not completely return to the positive electrode during discharge, leading to an upsurge in structural disorder and a shrinkage in the c-axis. It was ventured that lithium ions generated losses in the negative electrode or electrolyte and by the comparison of the two charging modules, c/a value was noticed to be the lowest when the CC-CV charging was 500 cycles, but did not affect the function of the positive electrode, indicating that the material was unwavering. The d_002_ of ideal graphite was 3.354 Å and after cyclic charging and discharging of the original graphite electrode, the interlayer spacing of d_002_ approached ideal value, especially in sine wave charging method. However, as the number of charges and discharges increased, the distance between the electrode d_002_ layers under both charging methods digressed from the ideal value, resulting in a more severe deviation from the constant voltage charging method.

Raman spectroscopy illustrated the Raman peak shift of positive electrode, signifying that LiCoO_2_ material endured structural modifications due to stress, and the peak intensities of A_1g_ and E_g_ modes significantly decreased with increasing charging and discharging times. It was also assumed that the decrease in characteristic peaks and the transmittance of lithium cobalt oxide material irradiated by laser light were due to the strong electrolyte signal, thus increasing light absorption coefficient and lessening the peak intensity. This may be due to lack of lithium ions in the positive electrode material. The graphite electrode in Raman spectroscopy exhibited the highest defect peak intensity after 500 cycles in CC-CV charging method, indicating disordered graphene stacking/damage to structural edges of graphite electrode. The degree of graphite defects increased with a less cycle numbers, indicating that the structure will automatically repair after the cycles. Under sine wave charging method for 100 cycles, the I_D_/I_G_ ratio was higher than the other method, but the increase in amplitude and defect ratio were significantly lower with escalating cycle times. The integrity of graphite structure stacking did not significantly differ with number of charges and discharges and modules. Inclusively, after SEM, XRD, and Raman electrode detections, it was determined that the structure of the positive electrode lithium cobalt oxide material of the battery was stable, and the changes in the two charging methods were inconsequential after 500 cycles. However, the damage to the graphite electrode in CC-CV charging method was more disadvantageous due to SEI film development**. Conclusions**.

This study is an investigation to determine the impacts of battery cycling charge and discharge on the electrodes under two different charging modules, and the two commercially successful charging methods, CC-CV and CC-Sinusoidal reflex, were implemented. As the number of charging and discharging cycles of the battery increased, the capacitance also had an initial slight increase followed by a gradual decrease, which initiated battery aging. Principally when CC-CV charging method was adopted, the trend of decreasing capacitance was much obvious. The conclusions of this study are as follows.

1. The difference between the positive electrode of lithium cobalt electrode and graphite electrode batteries was not significant from the SEM image, which may be due to the stable structure of lithium cobalt oxide positive electrode material. It was unaffected by the charging methods and cycle times.
2. According to EDS, the F element gradually combined with lithium ions during the charging and discharging process to form lithium fluoride, which was deposited on the surface of the negative electrode as one of the components of the SEI film.
3. The positive electrodes had an activation effect after less cycles of charging and discharging and after 300 or 500 cycles, the c/a value downfall designated that lithium ions may experience losses in the negative electrode or electrolyte.
4. The d_002_ of ideal graphite was3.354 Å, but as the charge and discharge cycles increased, the distance between the electrode d_002_ layers under both charging methods swerved from the ideal value, resulting in a more severe deviation from the CC-CV charging method.
5. Raman spectroscopy displayed the displacement of the Raman peak of the positive electrode, suggesting that the lithium cobalt oxide material might have underwent structural changes due to stress, and the peak intensified of A_1g_ and E_g_ modes significantly declined with increasing charging and discharging times. It was also observed that the characteristic peak values may drop due to the strong electrolyte signal compared to each other.
6. Raman spectroscopy detected that the graphite electrode had the highest defect peak intensity after 500 cycles in CC-CV charging method, indicating disordered graphene stacking or damage to the structural edges. The I_D_/I_G_ ratio of the CC-Sinusoid charging method was greater than that of the CC-CV charging method, but the increase in amplitude and defect ratio were significantly lower with more cycles. The integrity of graphite structure stacking had no significant variance with the number of charges and discharges and modules.
7. After SEM, XRD, and Raman detection, it was interestingly notified that the structure of the positive electrode lithium cobalt oxide material of the battery was stable, and the changes in the two charging methods were insignificant under 500 cycles. However, the damage to the negative graphite electrode with CC-CV charging was more deteriorating due to SEI film formations. Sinusoidal wave charging method was better for the electrodes.

## Data availability statement

The authors have chosen not to specify which data has been used.

## CRediT authorship contribution statement

**Huang K. David:** Writing – review & editing, Formal analysis. **Po-Tuan Chen:** Writing – original draft, Visualization, Validation, Methodology, Investigation, Formal analysis, Conceptualization. **Wei-Mon Yan:** Writing – review & editing, Resources, Project administration, Funding acquisition, Formal analysis. **Thangavel Sangeetha:** Writing – review & editing, Writing – original draft, Validation, Methodology, Data curation. **Cheng-Jung Yang:** Writing – review & editing, Writing – original draft, Supervision, Methodology, Funding acquisition, Formal analysis, Data curation, Conceptualization.

## Declaration of competing interest

The authors declare that they have no known competing financial interests or conflicts that could have influenced the research study reported in this paper.
