# Supplementary material for: Perspective of material evolution Induced by sinusoidal reflex charging in lithium-ion batteries
Source: Heliyon. 2024 May 4;10(10):e30471. doi: 10.1016/j.heliyon.2024.e30471 (PMC11096976; doi:10.1016/j.heliyon.2024.e30471)

**Perspective of Material Evolution Induced by Sinusoidal Reflex Charging in Lithium-Ion Batteries**

Po-Tuan Chen ^1^, K. David Huang ^1^, Wei-Mon Yan ^2^, Sangeetha Thangavel ^2*^, Cheng-Jung Yang ^3*^

^1^ Department of Vehicle Engineering, National Taipei University of Technology, Taipei, Taiwan

^2^ Department of Energy and Refrigerating Air-Conditioning Engineering, National Taipei University of Technology, Taipei, Taiwan

^3^ Program in Interdisciplinary Studies, National Sun Yat-sen University, Kaohsiung, Taiwan

| 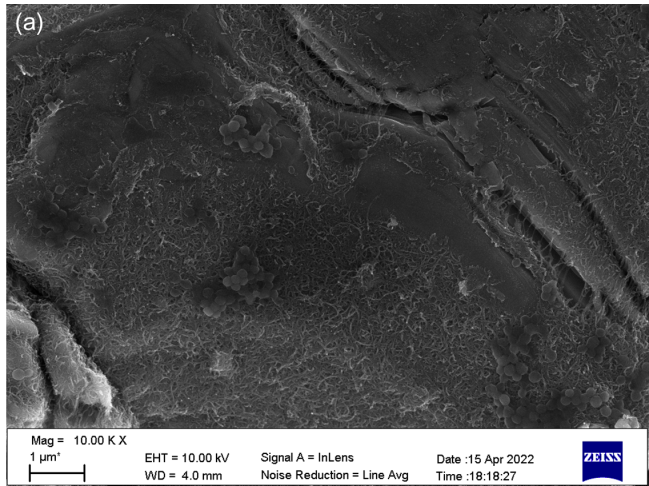 | 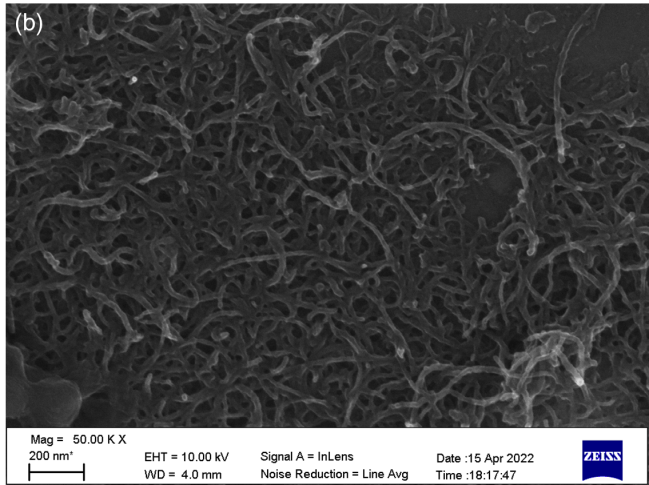 |
| --- | --- |
| 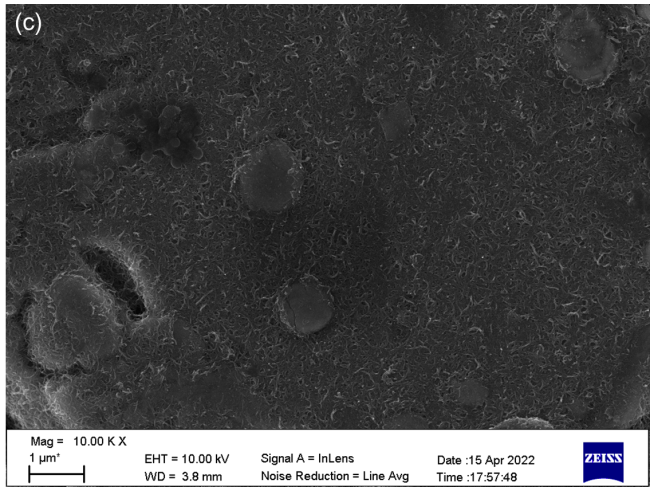 | 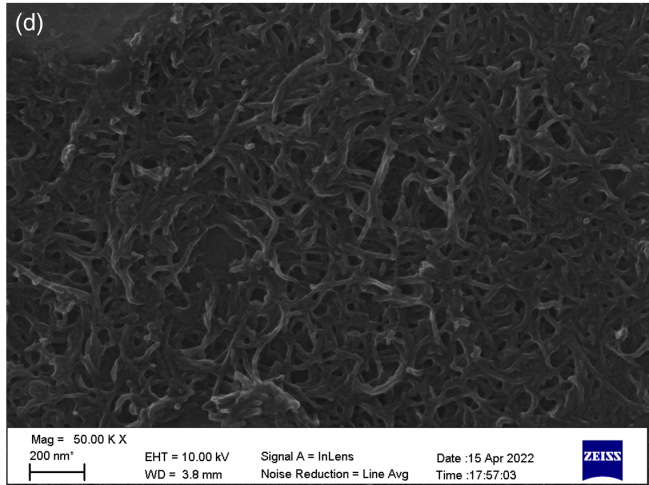 |
| 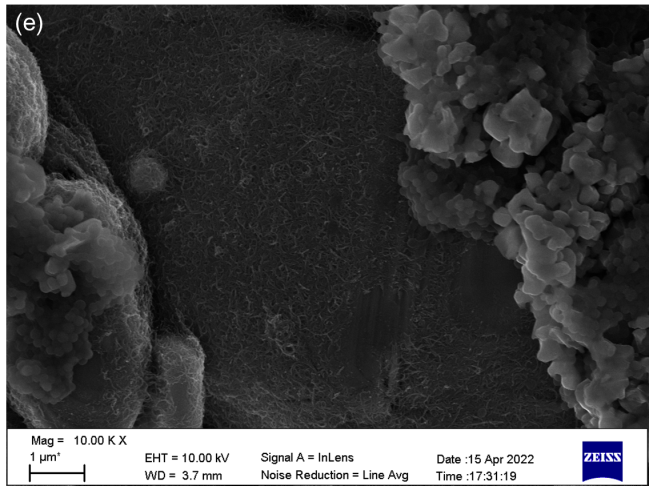 | 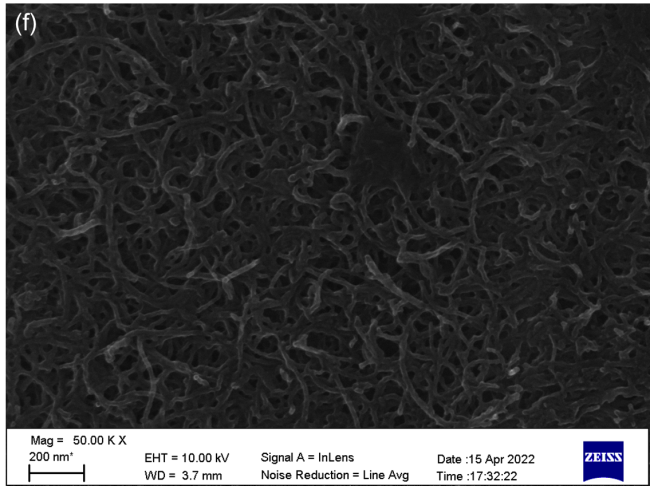 |

Figure S1. CC-CV charging and discharging cycles (a) 100 times of 10K magnification (b) 100 times of 50K magnification (c) 300 times of 10K magnification (d) 300 times of 50K magnification (e) 500 times of 10K magnification (f) 500 times of 50K magnification.

| 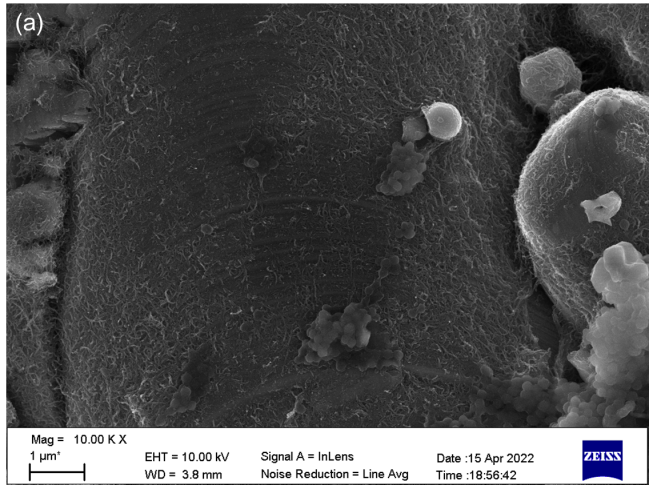 | 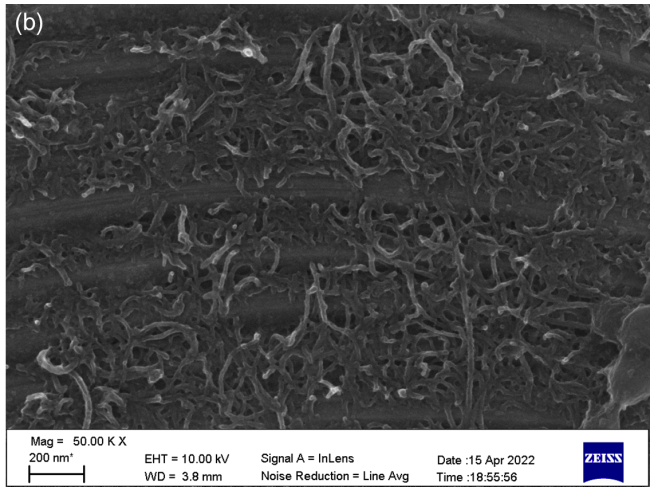 |
| --- | --- |
| 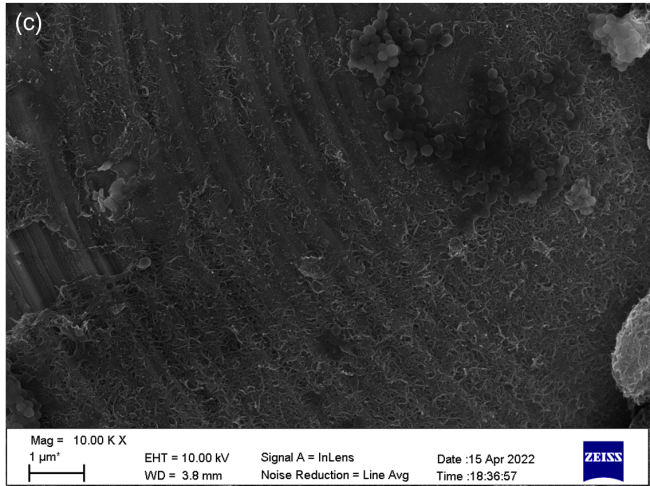 | 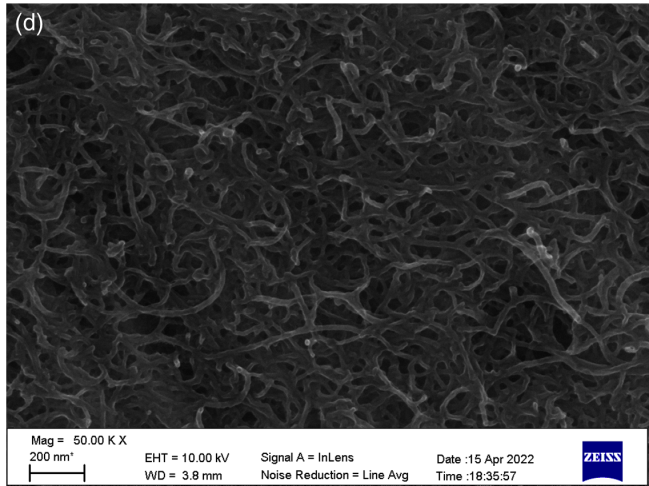 |
| 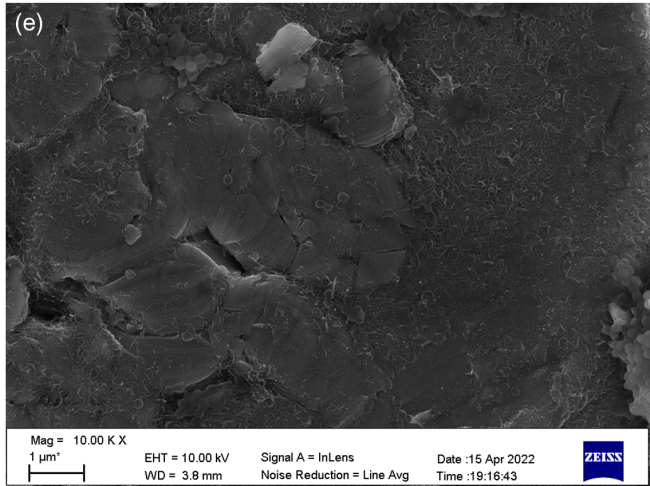 | 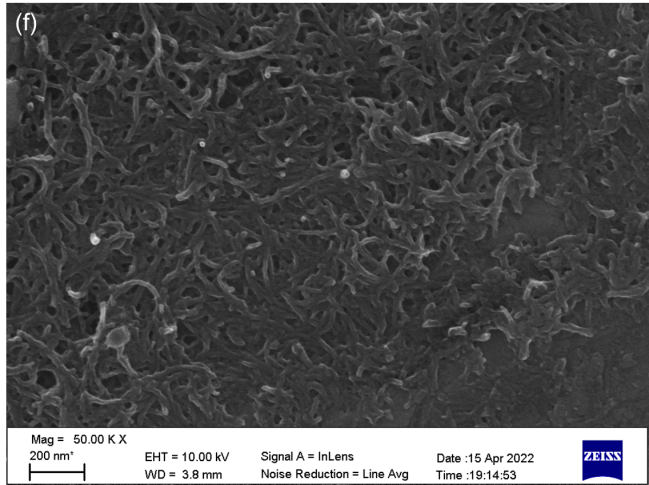 |

Figure S2. CC-Sinusoid charge and discharge cycle (a) 100 times 10K magnification (b) 100 times 50K magnification (c) 300 times 10K magnification (d) 300 times 50K magnification (e) 500 times 10K magnification (f) 500 times 50K magnification.

| 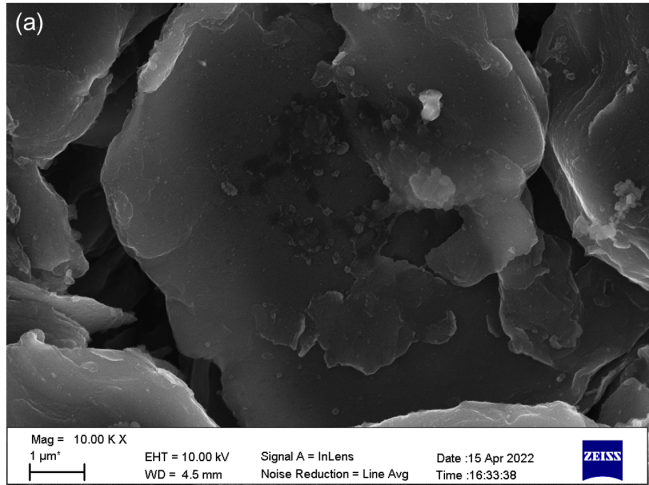 | 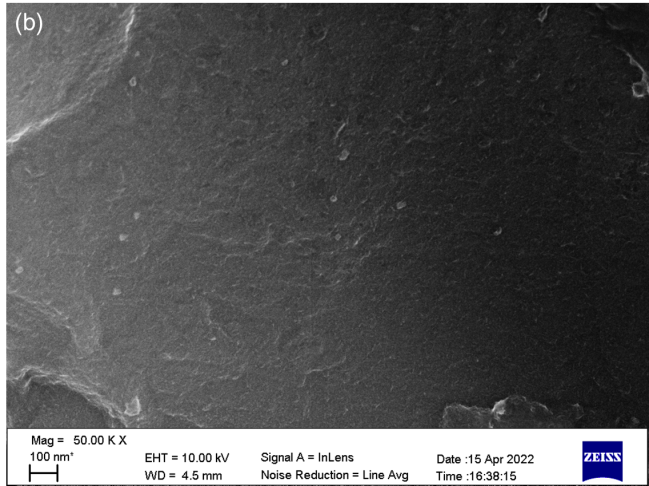 |
| --- | --- |
| 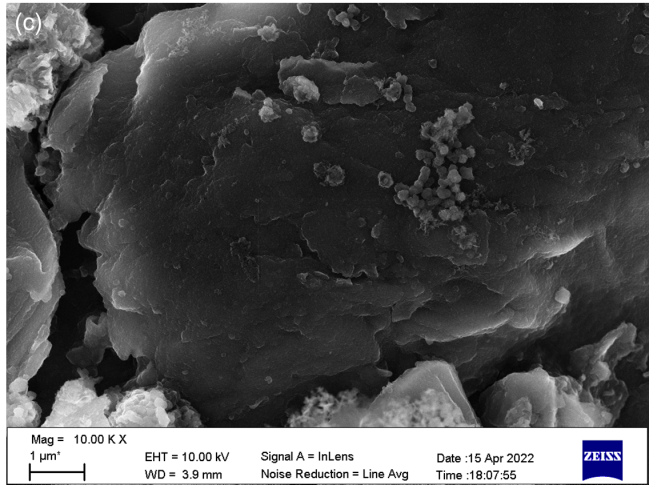 | 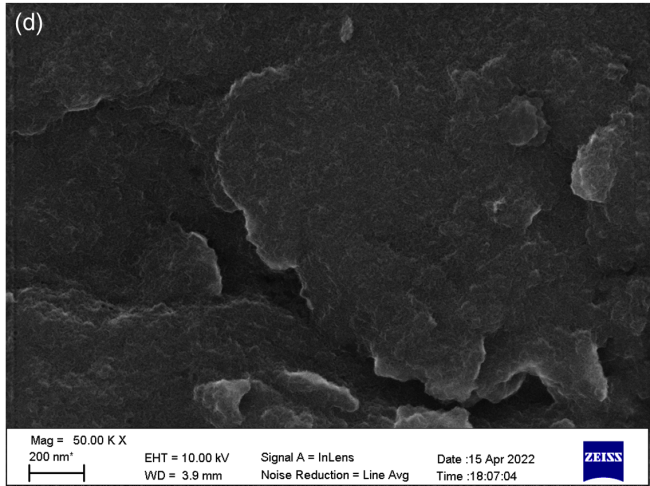 |
| 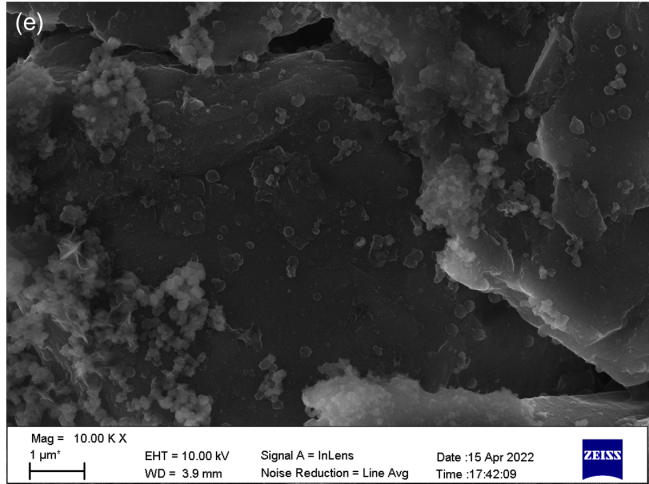 | 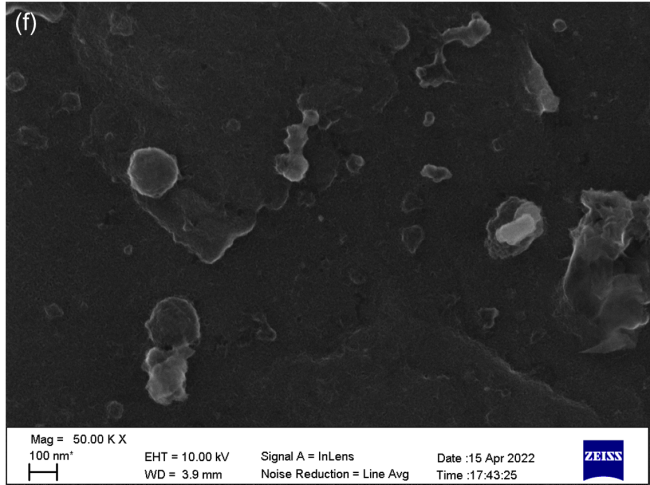 |
| 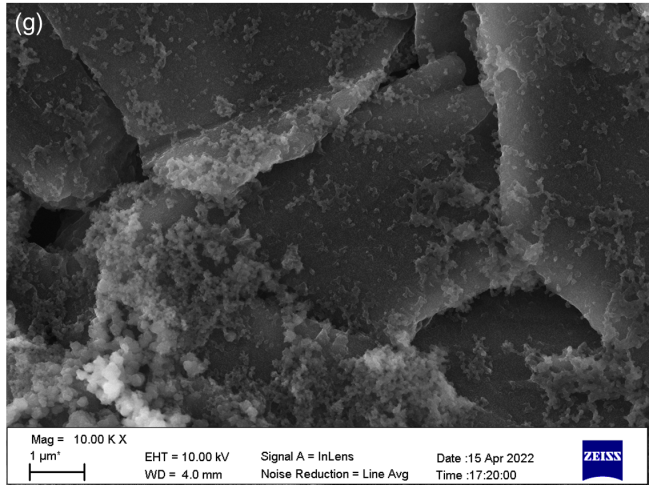 | 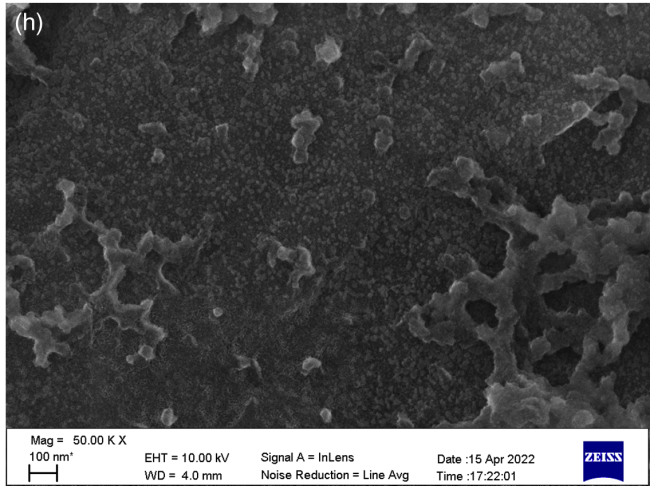 |

Figure S3. CC-CV charging and discharging cycle (a) Original amplification of 10K magnification (b) Original amplification of 50K magnification (c) 100th amplification of 10K magnification (d) 100th amplification of 50K magnification (e) 300th amplification of 10K magnification (f) 300th amplification of 50K magnification (g) 500th amplification of 10K magnification (h) 500th amplification of 50K magnification.

| 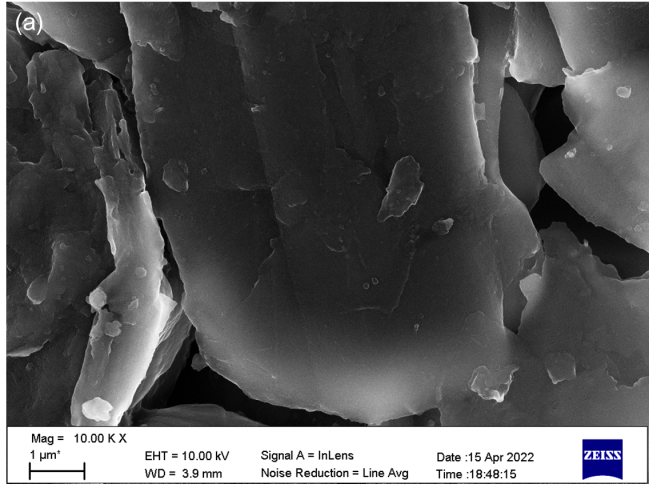 | 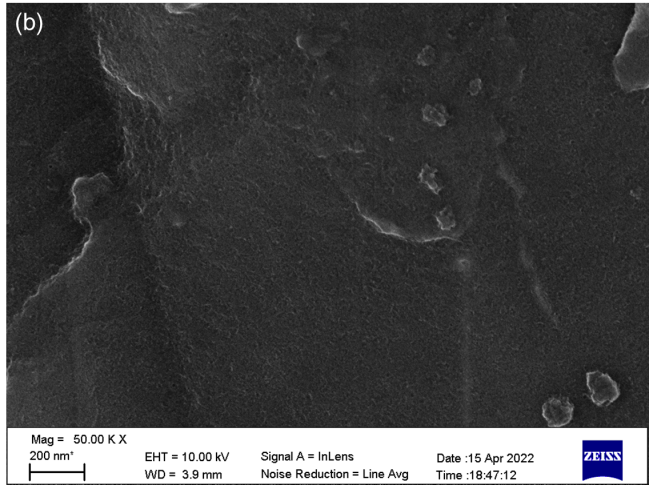 |
| --- | --- |
| 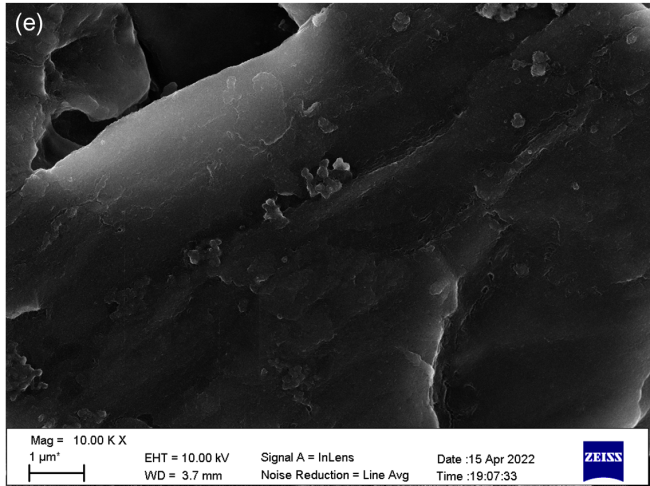 | 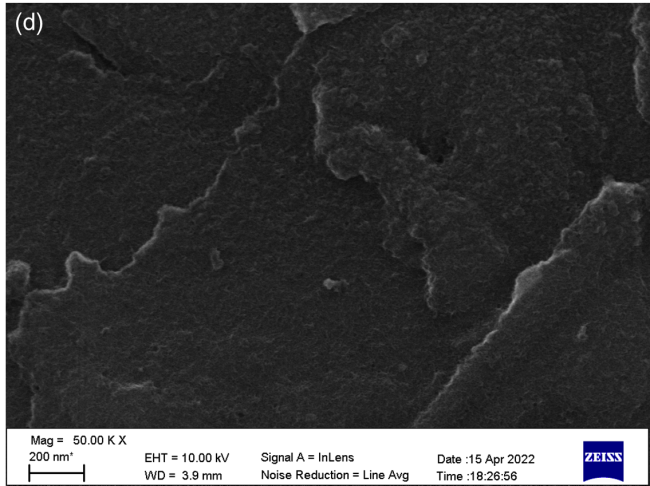 |
| 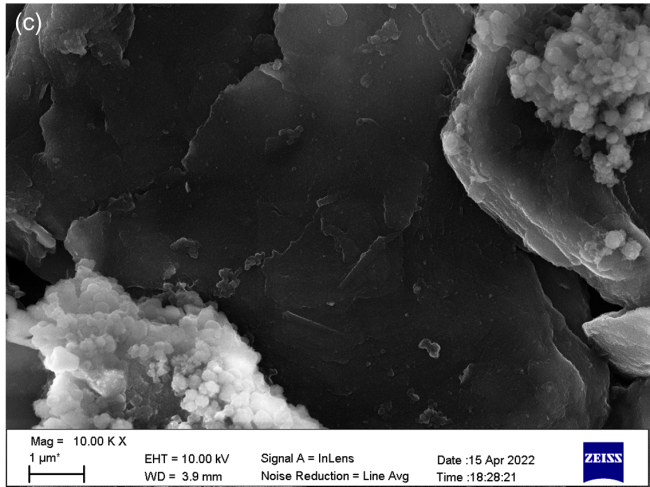 | 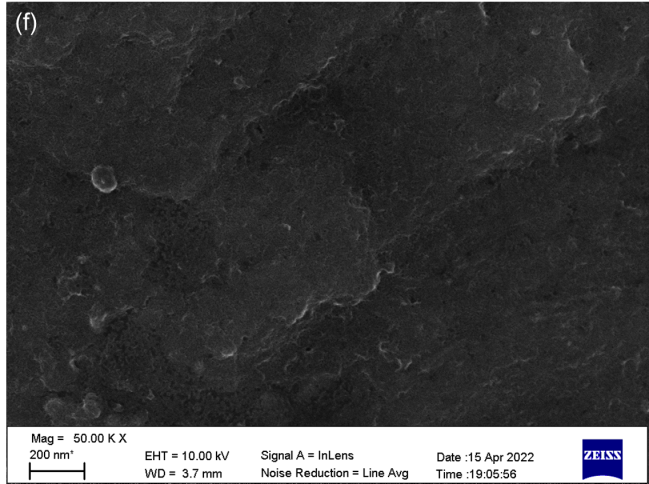 |

Figure S4. CC-Sinusoid charge discharge cycle (a) 100 times amplification of 10K magnification (b) 100 times amplification of 50K magnification (c) 300 times amplification of 10K magnification (d) 300 times amplification of 50K magnification (e) 500 times amplification of 10K magnification (f) 500 times amplification of 50K magnification

Table S1. The characteristic peak positions and crystal plane spacing of different charging and discharging cycles for the positive electrode under two different charging modules.


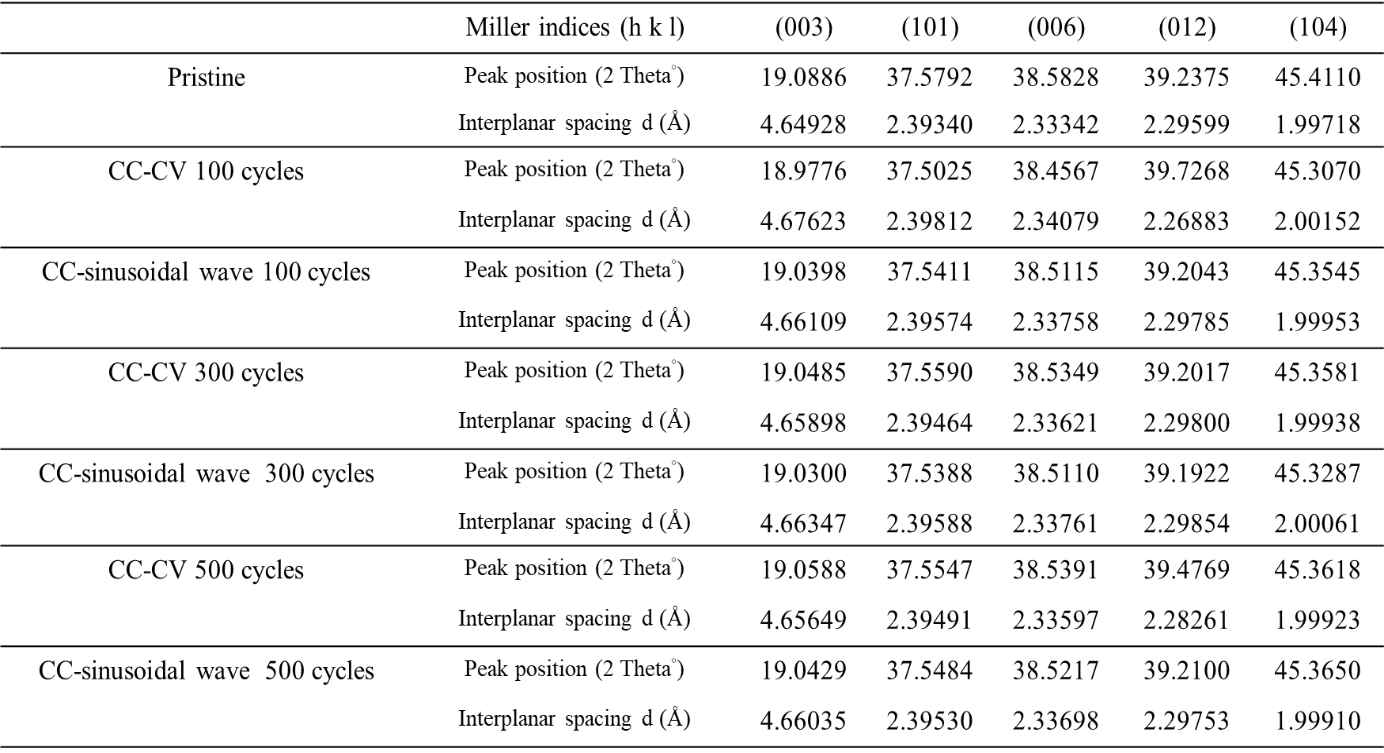


Table S2. Lattice constants and layer spacing for different charging and discharging cycles of the positive pole under two charging modules
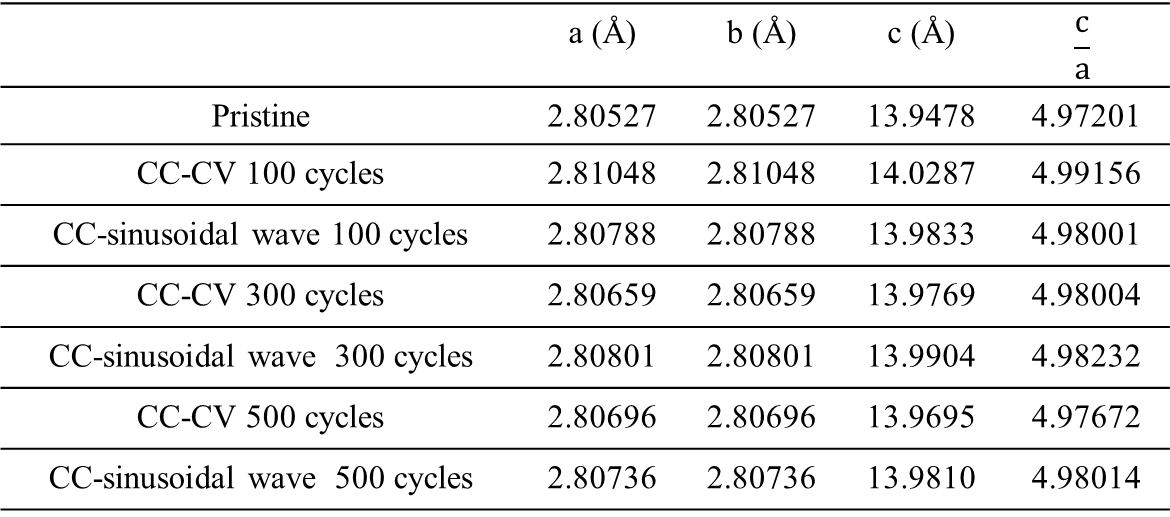


Table S3. The half width and crystal size of the main characteristic peak diffraction surface on the positive electrode


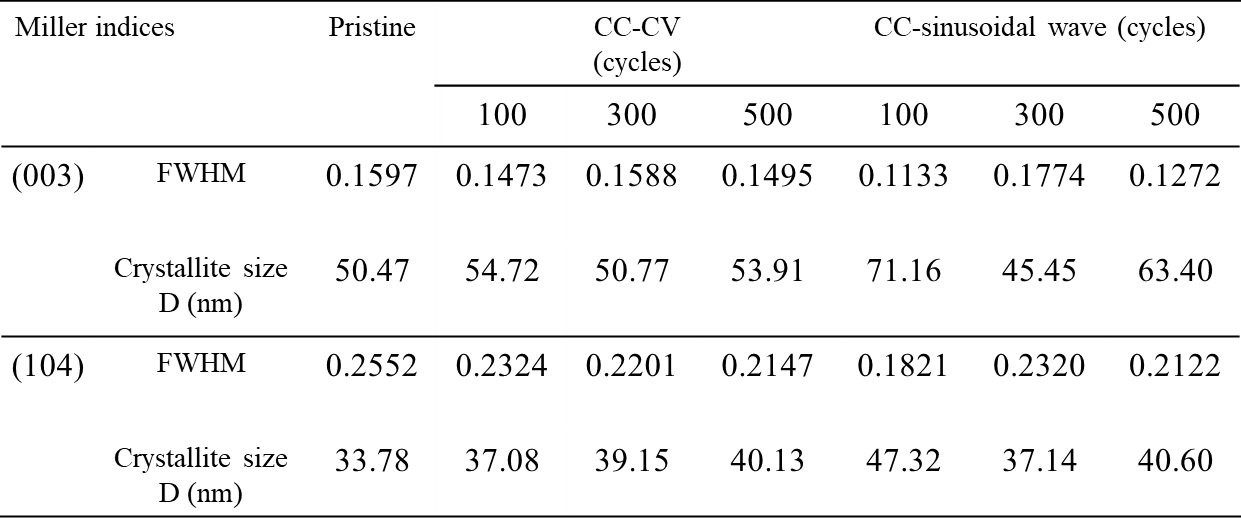


Table S4. The characteristic peak positions and crystal plane spacing of different charging and discharging cycles for the negative electrode under two different charging modules


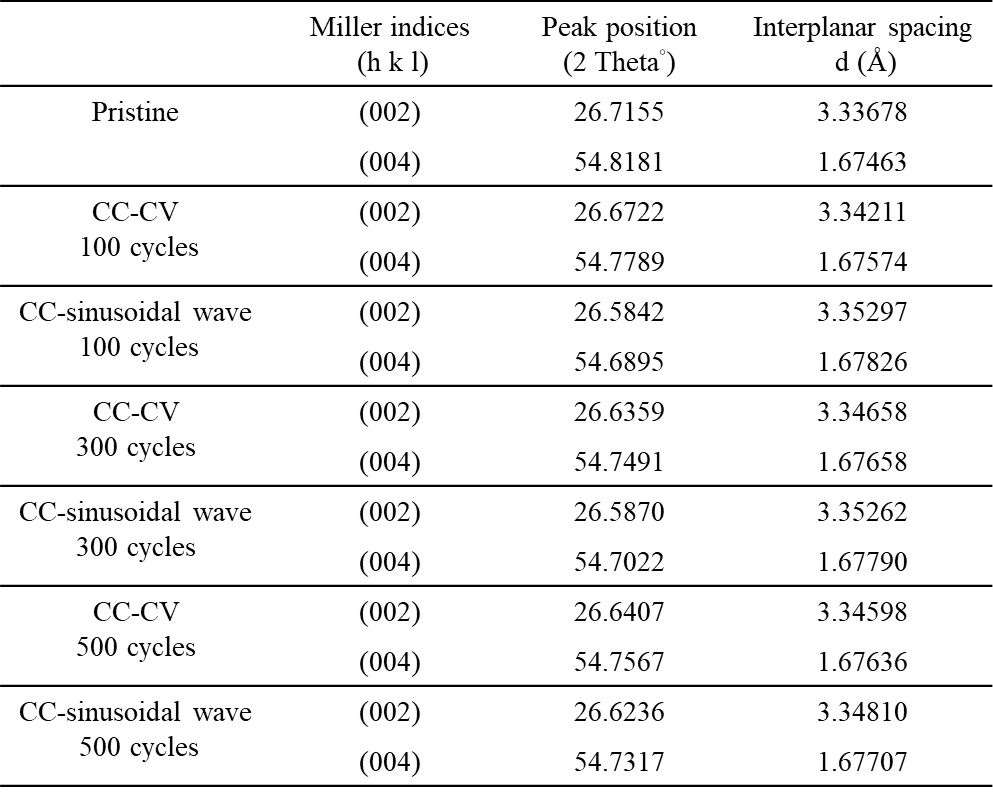


Table S5. Main Characteristic Peak Diffraction Faces, Halfwidth and Crystal Size of the Negative Electrode


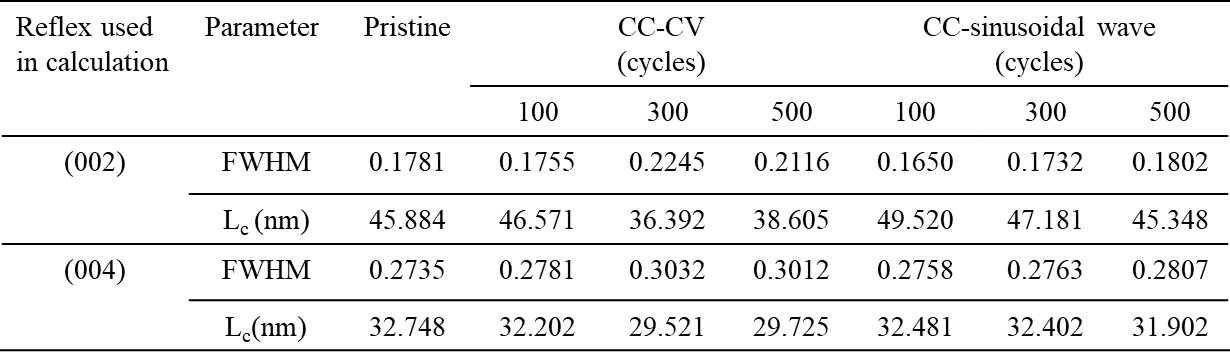

Supplement: Multimedia component 1 [file mmc1.docx]
